# A multi‐institutional retrospective study of carbon‐ion radiotherapy for non‐squamous cell malignant tumors of the nasopharynx: Subanalysis of Japan Carbon‐Ion Radiation Oncology Study Group study 1402 HN

**DOI:** 10.1002/cam4.1884

**Published:** 2018-11-18

**Authors:** Takanori Abe, Tatsuya Ohno, Masashi Koto, Yusuke Demizu, Hiroaki Suefuji, Hiroshi Tsuji, Tomoaki Okimoto, Yoshiyuki Shioyama, Jun‐ichi Saitoh, Katsuyuki Shirai, Kenji Nemoto, Takashi Nakano, Tadashi Kamada, Japan Carbon‐Ion Radiation Oncology Study Group, Hiroyuki Katoh

**Affiliations:** ^1^ Gunma University Heavy Ion Medical Center Maebashi Japan; ^2^ Hospital of the National Institute of Radiological Sciences, National Institutes for Quantum and Radiological Sciences and Technology Chiba Japan; ^3^ Department of Radiology Hyogo Ion Beam Medical Center Tatsuno Japan; ^4^ Ion Beam Therapy Center SAGA HIMAT Foundation Tosu Japan; ^5^ Department of Radiation Oncology Toyama University Toyama Japan; ^6^ Department of Radiology, Saitama Medical Center Jichi Medical University Saitama Japan; ^7^ Department of Radiation Oncology, Faculty of Medicine Yamagata University Yamagata Japan; ^8^ Department of Radiation Oncology Gunma University Graduate School of Medicine Maebashi Japan

**Keywords:** carbon‐ion radiotherapy, local control, non‐squamous cell malignant tumors of the nasopharynx, overall survival, toxicity

## Abstract

**Background:**

This multi‐institutional retrospective study focused on the clinical outcome of carbon‐ion radiotherapy (C‐ion RT) for non‐squamous cell malignant tumors of the nasopharynx.

**Methods:**

The Japan Carbon‐ion Radiation Oncology Study Group collected and analyzed data for 43 patients with non‐squamous cell malignant tumors of the nasopharynx treated with C‐ion RT at four institutions in Japan.

**Results:**

Twenty‐nine patients had adenoid cystic carcinomas, seven had malignant melanomas, three had adenocarcinomas, two had mucoepidermoid carcinomas, and two had other pathologies. Twenty‐six of the 43 patients (61%) had T4 tumors. The most common dose‐fractionation schedule was 64 Gy (relative biological effectiveness) in 16 fractions. The median follow‐up period was 30 months. The 2‐year local control (LC) and overall survival (OS) rates were 88% and 84%, respectively. For late toxicity, one patient developed grade 4 optic nerve disorder and two developed grade 5 pharyngeal hemorrhage. Actual incidence of grade 3 or higher late adverse events was 19%, and included cranial nerve dysfunction, jaw bone necrosis, central nervous system necrosis, and ear inflammation.

**Conclusions:**

C‐ion RT provided good LC and OS rates with acceptable toxicity for treatment of non‐squamous cell malignant tumors of the nasopharynx.

## 1 INTRODUCTION

Nasopharyngeal carcinoma (NPC) is common in South and Southeast Asia, where the incidence is 25 to 50 per 100 000 people.^1^ The most common histologic type of NPC is poorly differentiated squamous cell carcinoma (SCC), which is different from other head and neck cancers.^2^ As a result of its high radiosensitivity and complex anatomic location, standard treatment for nasopharyngeal SCC is concurrent chemoradiotherapy using X‐rays.^1^, ^2^, ^3^, ^4^ In contrast, non‐squamous cell malignant tumors of the nasopharynx such as adenoid cystic carcinoma, malignant melanoma, adenocarcinoma, and mucoepidermoid carcinoma are rare compared with SCC.^5^ In general, non‐squamous cell malignant tumors of the nasopharynx show resistance to X‐ray radiotherapy (RT) or chemotherapy.^6^ The refractoriness of non‐squamous cell malignant tumors of the nasopharynx means that there are no proper data for their treatment. Indeed, there are only a few single case reports about the clinical outcome of treatment for non‐squamous cell malignant tumors of the nasopharynx.^7^, ^8^, ^9^

Carbon‐ion RT (C‐ion RT) has been utilized since 1994 at the National Institute of Radiological Sciences in Japan to treat photon‐resistant tumors such as soft tissue sarcoma or malignant melanoma. C‐ion RT has shown favorable results by taking advantage of its higher relative biological effectiveness (RBE) and better dose concentration compared with those of X‐ray RT.^10^, ^11^ Theoretically, C‐ion RT has a potential advantage for non‐squamous cell malignant tumors of the nasopharynx in reducing toxicity by sparing critical organs adjacent to the nasopharynx and its higher probability of control of photon‐resistant tumors. Recently, there has been increasing evidence of its efficacy and safety for head and neck malignant tumors.^12^, ^13^, ^14^, ^15^, ^16^, ^17^, ^18^ However, to date, there are no reports of C‐ion RT for non‐squamous cell malignant tumors of the nasopharynx. Therefore, Japan Carbon‐ion Radiation Oncology Study Group (J‐CROS) which consisted of all of four institutions performing C‐ion RT in Japan undertook a study to assess the efficacy and safety of C‐ion RT for non‐squamous cell malignant tumors of the nasopharynx in a multi‐institutional retrospective analysis.

## 2 MATERIALS AND METHODS

### 2.1 Eligibility

This was a retrospective cohort study that included patients diagnosed with non‐squamous cell malignant tumors of the nasopharynx who had received C‐ion RT at four institutions in Japan, between November 2003 and December 2014. The four institutions were the Hospital of the National Institute of Radiological Sciences (Chiba, Japan), Gunma University Heavy Ion Medical Center (Maebashi, Japan), Hyogo Ion Beam Medical Center (Tatsuno, Japan), and Ion Beam Therapy Center, SAGA HIMAT Foundation (Tosu, Japan). Eligibility criteria were as follows: (a) histologically confirmed non‐squamous cell malignant tumors of the nasopharynx; (b) no bone or soft tissue tumors; (c) N0 or N1 and M0 status; (d) medically inoperable tumors or refusal of surgery; (e) treatment administered with definitive intent; (f) measurable tumors; and (g) Eastern Cooperative Oncology Group performance status of 0‐2. Patients with histology of SCC or who had previously received RT were excluded. This multi‐institutional retrospective study was approved by the Institutional Review Board of each institution (UMIN000024473).

### 2.2 Carbon‐ion RT

Dose of C‐ion RT was expressed in Gy (RBE), which was calculated by multiplying the physical dose of the C‐ion beam by an RBE of 3.^10^, ^11^ Prescribed total doses ranged from 57.6 Gy (RBE) to 70.4 Gy (RBE) and doses per fraction ranged from 2.2 Gy (RBE) to 4 Gy (RBE). Selection of dose‐fractionation was decided by each institution. Patients were immobilized using thermoplastic shells, and treatment planning computed tomography (CT) was performed. Contrast‐enhanced CT or magnetic resonance imaging (MRI) was undertaken concomitantly and fused in the treatment planning CT to help define gross tumor volume (GTV). The clinical target volume (CTV) margin, including microtumor invasion, was added to the GTV. The planning target volume (PTV) was defined as a summation of the CTV and 2‐5 mm of setup margin.

### 2.3 Follow‐up and evaluation

Acute toxicity was assessed daily during treatment. After treatment, diagnostic imaging such as CT, MRI, or fluoro‐deoxyglucose positron‐emission tomography/CT was performed every 2‐3 months for the first 2 years and every 3‐6 months thereafter. Acute and late adverse events were classified using the National Cancer Institute's Common Terminology Criteria for Adverse Events, version 4.0.^19^ Local recurrence was defined as recurrence in the irradiated field; progression was defined as local recurrence, lymph node recurrence, or distant metastasis; and overall survival (OS) was defined as the interval between initiation of C‐ion RT and the last follow‐up date when the patient was confirmed to be alive or the date when the patient died.

### 2.4 Statistical analysis

The local control (LC), progression‐free survival (PFS), and OS rates were calculated using the Kaplan‐Meier method and compared between subgroups using log‐rank test. Differences between groups were evaluated using *t* tests. For univariate analyses, log‐rank tests were used to compare LC and OS among the subgroups. Values of *P* < 0.05 were considered statistically significant, and all statistical tests were 2 sided. All statistical analyses were performed using IBM SPSS Statistics for Windows, version 23.0 (SPSS Inc, Armonk, NY, USA).

## 3 RESULTS

### 3.1 Patient characteristics

We retrospectively analyzed 43 patients with non‐squamous cell malignant tumors of the nasopharynx treated with C‐ion RT. Patient and tumor characteristics are summarized in Table 1. The median follow‐up period was 30 months (range, 3‐125 months). Concurrent chemotherapy with dacarbazine, nimustine, and vincristine was performed in three patients with malignant melanoma. Neoadjuvant chemotherapy was performed in three patients with adenocarcinoma, mucoepidermoid carcinoma, and adenoid cystic carcinoma.

**Table 1:** Patient and tumor characteristics (N = 43)

Characteristics	
Age, y, median (range)	63 (38‐76)
Sex, n (%)	
Male	13 (30)
Female	30 (70)
Performance status, n (%)	
0	23 (53)
1	20 (47)
Histo‐pathological type, n (%)	
Adenoid cystic carcinoma	29 (67)
Malignant melanoma	7 (16)
Adenocarcinoma	3 (7)
Mucoepidermoid carcinoma	2 (5)
Others	2 (5)
Disease, n (%)	
Primary tumor	38 (88)
Recurrent tumor	5 (12)
Operability, n (%)	
Operable	5 (12)
Inoperable	38 (88)
Combined therapy, n (%)	
Radiotherapy alone	37 (86)
Neoadjuvant chemotherapy	3 (7)
Concurrent chemotherapy	3 (7)
T classification, n (%)	
T1	1 (2)
T2	10 (23)
T3	6 (14)
T4	26 (61)
N classification, n (%)	
N0	40 (93)
N1	3 (7)
Gross tumor volume, cm^3^, median (range)	30 (3‐171)
Radiation dose, n (%)	
64 Gy (RBE) in 16 fractions	16 (37)
57.6 Gy (RBE) in 16 fractions	10 (23)
65 Gy (RBE) in 26 fractions	7 (16)
70.4 Gy (RBE) in 32 fractions	6 (14)
70.2 Gy (RBE) in 26 fractions	3 (7)
60.8 Gy (RBE) in 16 fractions	1 (2)

### 3.2 LC and survival

**Figure 1:**
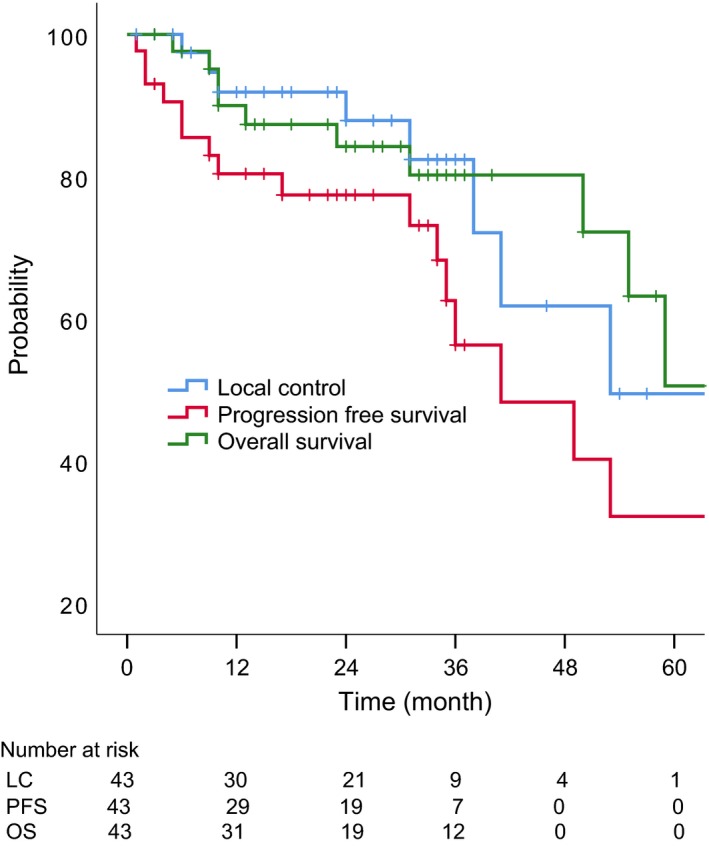
Local control, progression‐free survival, and overall survival for all the patients (N = 43)

Nine patients experienced local recurrence and one experienced lymph node recurrence. Eight patients died of primary disease, two of treatment‐related adverse events and one of intercurrent disease. The 2‐year estimated LC, PFS, and OS rates were 88%, 69%, and 84%, respectively. The LC, PFS, and OS curves are shown in Figure 1. In univariate analysis, there was no significant factor for LC, but sex, performance status, and T4 classification were significant factors for OS. These results are summarized in Table 2.

**Table 2:** Univariate analysis for LC and OS rates

	No. of` patients	2‐y LC (%)		2‐y OS (%)	
			*P* value		*P* value
Age					
<63	20	94	0.989	82	0.793
≳63	23	83		86	
Sex					
Male	13	73	0.134	63	<0.001
Female	30	93		93	
Performance status					
0	23	89	0.166	90	0.004
1	20	85		79	
Histology					
Adenoid cystic carcinoma	29	92	0.259	83	0.893
Malignant melanoma	7	67		86	
Adenocarcinoma	3	100		67	
Mucoepidermoid carcinoma	2	100		100	
Others	2	100		100	
T classification					
T1‐3	26	86	0.393	77	0.030
T4	17	91		94	
Operability					
Operable	5	86	0.192	81	0.489
Inoperable	38	100		80	
Tumor volume					
<30 cm^3^	22	80	0.770	95	0.064
≳30 cm^3^	21	100		73	
Radiation dose					
57.6 Gy (RBE)	10	88	0.064	89	0.962
≳64 Gy (RBE)	33	88		83	
Fractionation					
≤16 fractions	27	87	0.083	87	0.309
>16 fractions	16	83		80	

LC, local control; OS, overall survival; RBE, relative biological effectiveness.

### 3.3 Toxicity

Acute grade 3 mucositis was observed in nine patients (20%), and acute hematologic toxicity such as grade 3 leukopenia and anemia was observed in five patients (10%). No other acute toxicity of grade 3 or higher was observed. These acute symptoms were immediately resolved with conservative treatment. The late adverse events are summarized in Table 3. There were two patients with grade 5 pharyngeal hemorrhage and one with grade 4 optic nerve disorder. Fatal hemorrhage occurred in two adenoid cystic carcinoma patients with T4 tumors (tumor volume: 37 and 43 cm^3^) at 9 months and 14 months after treatment. Both of the tumors surrounded the carotid artery at diagnosis. The bleeding was caused by ulceration at the tumor site with tumor shrinkage in response to C‐ion RT.

**Table 3:** Late adverse events (N = 43)

	Grade 2	Grade 3	Grade 4	Grade 5
Mucositis	1 (2)	0 (0)	0 (0)	0 (0)
Osteonecrosis of jaw	4 (9)	1 (2)	0 (0)	0 (0)
Central nervous system necrosis	1 (2)	1 (2)	0 (0)	0 (0)
Pharyngeal hemorrhage	0 (0)	1 (2)	0 (0)	2 (4)
Hearing impairment	2 (4)	0 (0)	0 (0)	0 (0)
Optic nerve disorder	0 (0)	0 (0)	1 (2)	0 (0)
Cranial nerve disorder	2 (4)	1 (2)	0 (0)	0 (0)
Ear inflammation	5 (11)	2 (4)	0 (0)	0 (0)
Tinnitus	1 (2)	0 (0)	0 (0)	0 (0)
Trismus	1 (2)	0 (0)	0 (0)	0 (0)

Data are shown as n (%).

Five patients developed grade 2 or 3 osteonecrosis of jaw and seven developed grade 2 or 3 ear inflammation. Actual incidence of grade 3 or higher late adverse events was 19% (eight patients). Student's *t* test showed that mean tumor volume and age did not differ significantly between patients with or without grade 3 or higher late adverse events. The log‐rank test showed that T classification (T1‐3 or T4), histology (ACC or others), operability, dose‐fractionation (≤16 fractions or >16 fractions), and performance status (PS = 0 or 1) were not significantly correlated with occurrence of grade 3 or higher late adverse events.

## 4 DISCUSSION

In this retrospective analysis, the 2‐year estimated LC and OS rates were 88% and 84%, respectively. There was no significant factor for LC, which means that C‐ion RT for non‐squamous cell malignant tumors of the nasopharynx showed good efficacy regardless of histologic type, T stage, and other factors. There were only a few reports of site‐specific clinical outcome of C‐ion RT for head and neck malignant tumors. Hayashi et al^17^ reported that the 3‐year LC and OS were 81% and 94%, respectively, for 69 patients with major salivary gland carcinomas. Koto et al^18^ reported that the 2‐year LC and OS were 84% and 80%, respectively, for 458 patients with locally advanced sinonasal malignant tumors. Our study suggested that outcome of C‐ion RT for non‐squamous cell malignant tumors of the nasopharynx was comparable to that for other head and neck tumors, even though most of our patients had highly malignant disease such as inoperable advanced tumor and radioresistant histology.

It is frequently difficult to balance safety and efficacy of RT for locally advanced NPC compared with other head and neck tumors, in terms of sparing critical normal tissue such as brain, brain stem, optic nerve, and optic chiasm. In the present study, actual incidence of grade 3 or higher late adverse events with C‐ion RT was 19%, and the most common were ear inflammation and pharyngeal hemorrhage. Previous studies have shown that incidence of grade 3 or higher late adverse events such as ear inflammation, nervous system necrosis, and trismus ranged from 13% to 27% in X‐ray RT, with or without chemotherapy for nasopharyngeal SCC.^20^, ^21^, ^22^, ^23^ Toxicity profile in our study was comparable with the reported incidence of late adverse events after X‐ray RT for nasopharyngeal SCC. We conclude that C‐ion RT for non‐squamous cell malignant tumors of the nasopharynx shows promising efficacy with acceptable toxicity.

Fatal adverse events should be carefully discussed in detail, because, even if the incidence is not high, two patients with adenoid cystic carcinoma (4%) developed fatal pharyngeal hemorrhage. Patients with nasopharyngeal tumors surrounding the carotid artery should be carefully followed during and after C‐ion RT. In our study, grade 4 visual impairment was observed in one patient (2%). In this case, T4 tumor invaded the orbital space and was close to the optic nerve, which made it difficult to reduce the dose to the optic nerve while delivering an adequate dose to the tumor. In all patients with highly advanced tumor who were expected to develop high‐grade adverse events, careful and adequate explanation was given repeatedly and informed consent was obtained prior to the treatment. Recently, much effort has been made to reduce adverse events.^24^, ^25^, ^26^, ^27^, ^28^, ^29^ For example, we identified the optimal cutoff dose constraints for predicting the occurrence of brain necrosis by analysis of past cases and the dose constraints might help minimize brainstem necrosis after C‐ion RT.^24^ We have shared these experiences within J‐CROS and all institutions treat patients with unified dose‐fractionation schedules and accumulate their clinical results to clarify the efficacy and toxicity profiles of C‐ion RT for non‐squamous cell malignant tumors of the nasopharynx.

This study had some limitations. First, this was a retrospective study with a small number of patients. However, because of the lack of clinical results about non‐squamous cell malignant tumors of the nasopharynx, we think that this study provides beneficial clinical information for treatment of these tumors. Second, further follow‐up is necessary to confirm the long‐term efficacy and incidence of late toxicity. Third, there was heterogeneity regarding the dose‐fractionation schedules because this study retrospectively analyzed the data collected from four institutions. To overcome these limitations, we have been conducting a multi‐institutional registry study using fixed dose‐fractionation schedules such as 64 Gy(RBE) in 16 fractions or 57.6 Gy(RBE) in 16 fractions for the tumor which widely involves mucosa.

In conclusion, this multi‐institutional retrospective study showed that C‐ion RT achieved good LC and OS rates, with acceptable toxicity for non‐squamous cell malignant tumors of the nasopharynx.

## CONFLICT OF INTEREST

The authors have no conflict of interest.

## APPENDIX 1 Japan Carbon‐ion Radiation Oncology Study Group

Japan Carbon‐ion Radiation Oncology Study Group is consisted of Hospital of the National Institute of Radiological Sciences, National Institutes for Quantum and Radiological Sciences and Technology, Chiba, Japan (Masashi Koto: koto.masashi@qst.go.jp); Gunma University Heavy Ion Medical Center, Maebashi, Japan (Tatsuya Ohno: tohno@gunma-u.ac.jp); Department of Radiology, Hyogo Ion Beam Medical Center, Tatsuno, Japan (Tomoaki Okimoto: kiamoto935@gmail.com); Ion Beam Therapy Center, SAGA HIMAT Foundation, Tosu, Japan (Yoshiyuki Shioyama: shioyama-yoshiyuki@saga-himat.jp); Kanagawa Cancer Center, Yokohama, Japan (Hiroyuki Katoh: hkatoh@kcch.jp)

## References

[1] Kamran SC , Riaz N , Lee N . Nasopharyngeal carcinoma. Surg Oncol Clin N Am. 2015;24:547‐561. 25979399 10.1016/j.soc.2015.03.008

[2] Lee AW , Ng WT , Chan YH , et al. The battle against nasopharyngeal cancer. Radiother Oncol. 2012;104:272‐278. 22938727 10.1016/j.radonc.2012.08.001

[3] Chan AT . Nasopharyngeal carcinoma. Ann Oncol. 2010;21:308‐312. 10.1093/annonc/mdq277 20943634

[4] Lee AW , Ma BB , Ng WT , Chan AT . Management of nasopharyngeal carcinoma: current practice and future perspective. J Clin Oncol. 2015;33:3356‐3364. 26351355 10.1200/JCO.2015.60.9347

[5] Fletcher C . Diagnostic Histopathology of Tumors, 4th edn Amsterdam, The Netherlands: Elsevier Saunders; 2013.

[6] Joiner M , van del Kogel A . Basic Clinical Radiobiology. Oxford, UK: CRC Press; 2009.

[7] Elkholti Y , Cosmidis A , Ardiet JM , Laffay L , De Bari B . Adenoid cystic carcinoma of the nasopharynx: a case report and a discussion about prognostic factors and the role of local treatments. Tumori. 2013;99:55‐60. 10.1177/030089161309900229 23748830

[8] Gentile MS , Yip D , Liebsch NJ , et al. Definitive proton beam therapy for adenoid cystic carcinoma of the nasopharynx involving the base of skull. Oral Oncol. 2017;65:38‐44. 28109466 10.1016/j.oraloncology.2016.11.016

[9] Phan J , Ng SP , Courtney Pollard I , Phan J . A rare case of unresectable adenoid cystic carcinoma of the nasopharynx treated with intensity modulated proton therapy. Cureus. 2017;9:1688. 10.7759/cureus.1688 PMC5703592 29188150

[10] Ohno T . Particle radiotherapy with carbon ion beams. EPMA J. 2013;4:9. 23497542 10.1186/1878-5085-4-9 PMC3598788

[11] Kamada T . A review of update clinical results of carbon ion radiotherapy. Jpn J Clin Oncol. 2012;42:670‐685. 22798685 10.1093/jjco/hys104 PMC3405871

[12] Shirai K , Saitoh JI , Musha A , et al. Prospective observational study of carbon‐ion radiotherapy for non‐squamous cell carcinoma of the head and neck. Cancer Sci. 2017;108:2039‐2044. 28730646 10.1111/cas.13325 PMC5623744

[13] Saitoh JI , Koto M , Demizu Y , et al. A multicenter study of carbon‐ion radiation therapy for head and neck adenocarcinoma. Int J Radiat Oncol Biol Phys. 2017;99:442‐449. 28871995 10.1016/j.ijrobp.2017.04.032

[14] Shirai K , Koto M , Demizu Y , et al. Multi‐institutional retrospective study of mucoepidermoid carcinoma treated with carbon‐ion radiotherapy. Cancer Sci. 2017;108:1447‐1451. 28474791 10.1111/cas.13270 PMC5497800

[15] Koto M , Demizu Y , Saitoh JI , et al. Multicenter Study of Carbon‐Ion Radiation Therapy for Mucosal Melanoma of the Head and Neck: subanalysis of the Japan Carbon‐Ion Radiation Oncology Study Group (J‐CROS) Study (1402 HN). Int J Radiat Oncol Biol Phys. 2017;97:1054‐1060. 28332989 10.1016/j.ijrobp.2016.12.028

[16] Sulaiman NS , Demizu Y , Koto M , et al. Multicenter study of carbon‐ion radiation therapy for adenoid cystic carcinoma of the head and neck: subanalysis of the Japan Carbon‐Ion Radiation Oncology Study Group (J‐CROS) Study (1402 HN). Int J Radiat Oncol Biol Phys. 2018;100:639‐646. 29413278 10.1016/j.ijrobp.2017.11.010

[17] Hayashi K , Koto M , Demizu Y , et al. A retrospective multicenter study of carbon‐ion radiotherapy for major salivary gland carcinomas: subanalysis of J‐CROS 1402 HN. Cancer Sci. 2018;109:1576‐1582. 29493851 10.1111/cas.13558 PMC5980152

[18] Koto M , Demizu Y , Saitoh J , et al. Definitive carbon‐ion radiation therapy for locally advanced sinonasal malignant tumors: subgroup analysis of a multicenter study by the Japan Carbon‐Ion Radiation Oncology Study Group (J‐CROS). Int J Radiat Oncol Biol Phys. 2018;102:353‐361. 30191869 10.1016/j.ijrobp.2018.05.074

[19] US. Department of Health and Human Services , National Institutes of Health , National Cancer Institute . Common Terminology Criteria for Adverse Events v4.0. 2013 https://evs.nci.nih.gov/ftp1/CTCAE/CTCAE_4.03_2010-06-14_Quick Reference_5x7.pdf#search='NIH+NCI+CTCAE+4'. Accessed March 10, 2018.

[20] Chen QY , Wen YF , Guo L , et al. Concurrent chemoradiotherapy vs radiotherapy alone in stage II nasopharyngeal carcinoma: phase III randomized trial. J Natl Cancer Inst. 2011;103:1761‐1770. 22056739 10.1093/jnci/djr432

[21] Lee AW , Tung SY , Chua DT , et al. Randomized trial of radiotherapy plus concurrent‐adjuvant chemotherapy vs radiotherapy alone for regionally advanced nasopharyngeal carcinoma. J Natl Cancer Inst. 2010;102:1188‐1198. 20634482 10.1093/jnci/djq258

[22] Lin JC , Jan JS , Hsu CY , Jiang RS , Wang WY . Outpatient weekly neoadjuvant chemotherapy followed by radiotherapy for advanced nasopharyngeal carcinoma: high complete response and low toxicity rates. Br J Cancer. 2003;88:187‐194. 12610501 10.1038/sj.bjc.6600716 PMC2377053

[23] Liu YC , Wang WY , Twu CW , et al. Comparison long‐term outcome of definitive radiotherapy plus different chemotherapy schedules in patients with advanced nasopharyngeal carcinoma. Sci Rep. 2018;8:470. 29323141 10.1038/s41598-017-18713-z PMC5764995

[24] Shirai K , Fukata K , Adachi A , et al. Dose‐volume histogram analysis of brainstem necrosis in head and neck tumors treated using carbon‐ion radiotherapy. Radiother Oncol. 2017;125:36‐40. 28867558 10.1016/j.radonc.2017.08.014

[25] Musha A , Shimada H , Shirai K , et al. Prediction of acute radiation mucositis using an oral mucosal dose surface model in carbon ion radiotherapy for head and neck tumors. PLoS ONE. 2015;10:e0141734. 26512725 10.1371/journal.pone.0141734 PMC4626117

[26] Koto M , Hasegawa A , Takagi R , et al. Risk factors for brain injury after carbon ion radiotherapy for skull base tumors. Radiother Oncol. 2014;111:25‐29. 24332023 10.1016/j.radonc.2013.11.005

[27] Hasegawa A , Mizoe JE , Mizota A , Tsujii H . Outcomes of visual acuity in carbon ion radiotherapy: analysis of dose‐volume histograms and prognostic factors. Int J Radiat Oncol Biol Phys. 2006;64:396‐401. 16182466 10.1016/j.ijrobp.2005.07.298

[28] Sasahara G , Koto M , Ikawa H , et al. Effects of the dose‐volume relationship on and risk factors for maxillary osteoradionecrosis after carbon ion radiotherapy. Radiat Oncol. 2014;9:92. 24708583 10.1186/1748-717X-9-92 PMC3992144

[29] Musha A , Saitoh J‐I , Shirai K , et al. Customized mouthpieces designed to reduce tongue mucositis in carbon‐ion radiotherapy for tumors of the nasal and paranasal sinuses. Phys Imaging Radiat Oncol. 2017;3:1‐4.

